# Crystal structure of bis­(azido-κ*N*)bis­(quinolin-8-amine-κ^2^ *N*,*N*′)iron(II)

**DOI:** 10.1107/S2056989016014808

**Published:** 2016-09-30

**Authors:** Fatima Setifi, Dohyun Moon, Robeyns Koen, Zouaoui Setifi, Morad Lamsayah, Rachid Touzani

**Affiliations:** a Laboratoire de Chimie, Ingénierie Moléculaire et Nanostructures (LCIMN), Université Ferhat Abbas Sétif 1, Sétif 19000, Algeria; b Pohang Accelerator Laboratory, POSTECH, Pohang 37673, Republic of Korea; c Institute of Condensed Matter and Nanosciences (IMCN), Université Catholique de Louvain, 1 Place Louis Pasteur, B 1348 Louvain-la-Neuve, Belgium; d Laboratoire de Chimie Appliquée et Environnement, LCAE-URAC18, COSTE, Faculté des Sciences, Université Mohamed Premier, BP524, 60000, Oujda, Morocco; e Faculté Pluridisciplinaire Nador BP 300, Selouane, 62702, Nador, Morocco

**Keywords:** crystal structure, hydro­thermal synthesis, coordination compound, Fe^II^ complex, quinolin-8-amine, azide, hydrogen bonding, π–π stacking

## Abstract

The search for new mol­ecular materials with inter­esting magnetic properties using the pseudohalide azide ion and quinolin-8-amine (aqin, C_9_H_8_N_2_) as a chelating ligand, led to the synthesis and structure determination of the title complex, [Fe(N_3_)_2_(C_9_H_8_N_2_)_2_]. The complex shows an octa­hedral geometry, with the Fe^II^ atom surrounded by six N atoms; the two N_3_^−^ anions coordinate in a *cis* configuration, while the remaining N atoms originate from the two quinolin-8-amine ligands with the quinoline N atoms lying on opposite sides of the Fe atom. The crystal packing is dominated by layers of hydro­philic and aromatic regions parallel to the *ac* plane, stabilized by a two-dimensional hydrogen-bonded network and π–π stacking.

## Abstract 2

Exploring the role of azido anions led to the structure of this heteroleptic Fe complex with two azide ions and two quinolin-8-amine ligands.

## Chemical context   

In recent years, mol­ecular magnetism has attracted great attention due to the inter­est in designing new mol­ecular materials with inter­esting magnetic properties and potential applications (Kahn, 1993 ▸; Miller & Gatteschi, 2011 ▸). Connecting paramagnetic centers by use of bridging polynitrile or pseudohalide ligands is an important strategy to design such materials (Setifi *et al.*, 2002 ▸, 2003 ▸; Gaamoune *et al.*, 2010 ▸; Miyazaki *et al.*, 2003 ▸; Benmansour *et al.*, 2008 ▸, 2009 ▸; Yuste *et al.*, 2009 ▸; Setifi *et al.*, 2013 ▸, 2014 ▸; Addala *et al.*, 2015 ▸). As a short bridging ligand and efficient superexchange mediator, the pseudohalide azide ion has proved to be very versatile and diverse in both coordination chemistry and magnetism. It can link metal ions in μ-1,1 (end-on, EO), μ-1,3 (end-to-end, EE), μ-1,1,1 and other modes, and effectively mediate either ferromagnetic or anti­ferromagnetic coupling. Many azide-bridged systems with different dimensionality and topologies have been synthesized by using various auxiliary ligands, and a great diversity of magnetic behavior has been demonstrated (Ribas *et al.*, 1999 ▸; Gao *et al.*, 2004 ▸; Liu *et al.*, 2007 ▸; Mautner *et al.*, 2010 ▸). In view of the possible roles of the versatile azido ligand, we have been inter­ested in using it in combination with other chelating or bridging neutral co-ligands to explore their structural and electronic characteristics in the field of mol­ecular materials exhibiting inter­esting magnetic exchange coupling. During the course of attempts to prepare such complexes with quinolin-8-amine, we isolated the title compound, whose structure is described herein.
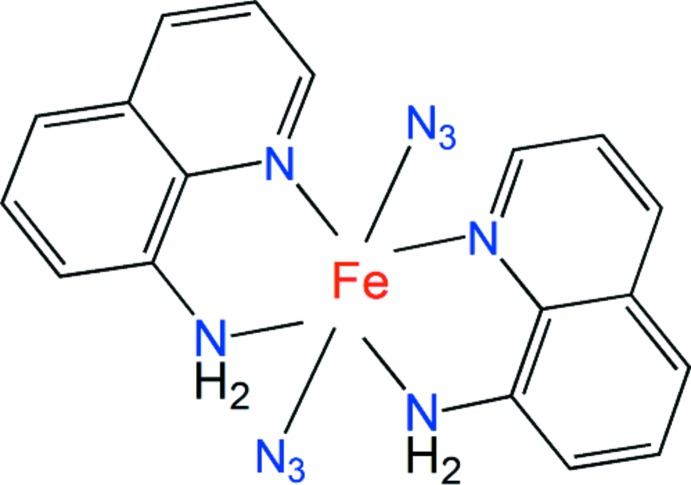


## Structural commentary   

**Figure 1:**
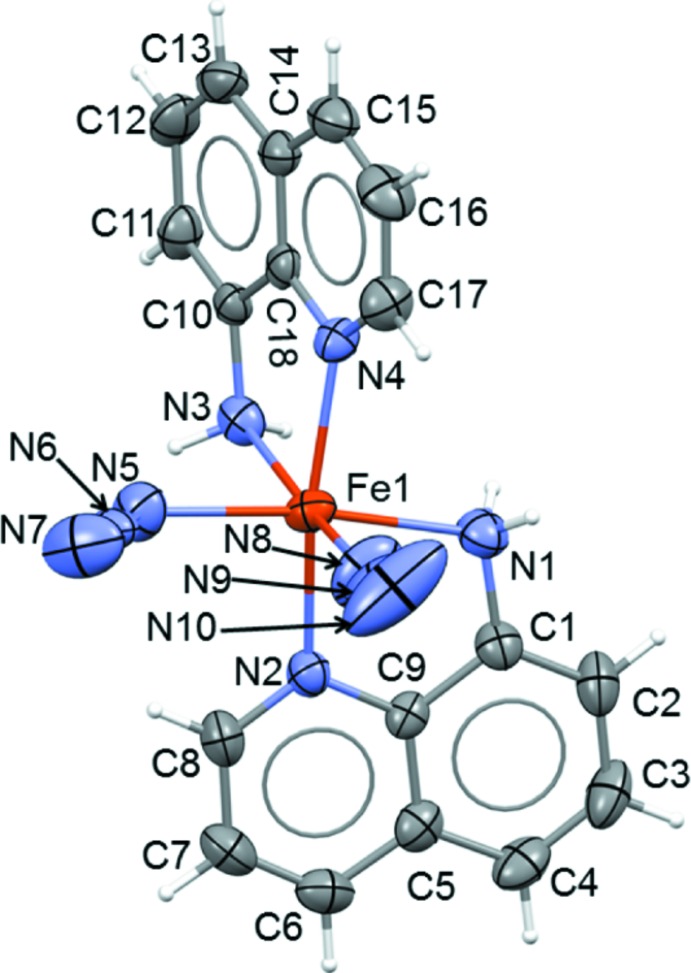
The mol­ecular structure of the title compound, showing the atom-labelling scheme. Displacement ellipsoids are drawn at the 50% probability level.

The title compound shows an octa­hedral coordination around the Fe^II^ atom. The Fe complex is a neutral and discrete mol­ecule and the two coordinating N_3_^−^ anions occupy adjacent sites, classifying the title compound as a *cis*-complex. Fig. 1 ▸ shows the mol­ecular structure.

The octa­hedral positions are occupied by six nitro­gen atoms where the quinoline aromatic nitro­gen atoms are found in the *trans* positions. All six Fe—N bond lengths are essentially uniform [2.104 (3)–2.284 (3) Å] and typical for high-spin iron(II) compounds (Table 1 ▸). The Fe—NH_2_ bond lengths are somewhat longer (∼0.10 Å) than the other Fe—N bonds. As a result of the quinolin-8-amine bite angle of about 75° the octa­hedral geometry is slightly distorted, allowing better separation of the negative charges on the azide ligands.

## Supra­molecular features   

**Figure 2:**
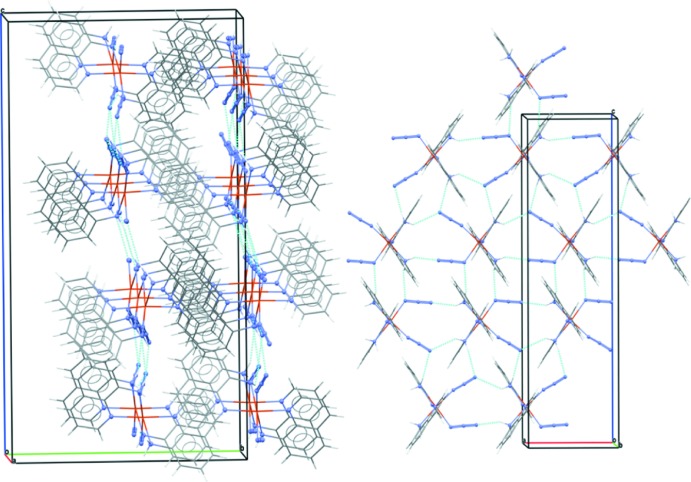
(Left) A view down the *a* axis, showing the alternating layers of hydro­philic and aromatic regions. (Right) The hydrogen-bonding network found in the hydro­philic region.

Looking down the *a* axis (Fig. 2 ▸) one can notice alternating layers (stacked along the *b*-axis direction) of hydro­philic and aromatic regions. This layering can also be seen at the level of the complex itself, where the aromatic quinoline moieties are located above and below the hydro­philic plane formed by the NH_2_ and N_3_^−^ groups. These latter are engaged in hydrogen bonds expanding along the *ac* plane (Table 2 ▸). Both H atoms of the NH_2_ group involving N1 form hydrogen bonds with the terminal nitro­gen atoms of two neighboring (symmetry-related) azide ligands. The other NH_2_ group has one of its hydrogen atoms (N3—N3*A*) involved in a similar inter­action, and the other hydrogen (N3—N3*B*) shows a very weak inter­action with the coordinating end of a neighboring azide ion. The aromatic rings on the other hand show parallel displaced π-stacking between pairs of quinoline (Q) moieties, the distance between the two quinoline planes is 3.38 Å (measured as the distance between the centroid of Q1 and the plane through Q2), or 3.35 Å, when inter­changing Q1 and Q2. Some of the hydrogen bonds (Table 2 ▸) are rather long and the stabilization of the crystal packing comes from the combined effect of the hydrogen-bonding inter­actions, which direct the orientation of the neighboring complexes and the additional π–π stacking inter­actions that hold the complexes in place.

## Database survey   

A search in the Cambridge Structural Database (Version 5.37, Feb 2016 with two updates; Groom *et al.*, 2016 ▸) reveals that only nine Fe^II^ complexes with quinolin-8-amine groups have been reported. None of these complexes involve azide groups, neither coordinating nor as a free anion. There is one known Cd complex that contains 8-amino­quinoline and bound azide; rather than forming discrete entities, the Cd complex is polymeric, expanding into chains where the azides act as bridging ligands [refcodes WIJWES (Paira *et al.*, 2007 ▸) and WIJWES01 (Xu *et al.*, 2008 ▸)] in the EO mode. Considering the azides and their coordination modes, the predominant N_3_^−^ binding mode is as monodentate (2210 entries), among the bridging modes the μ_2_ modes either 1,1 EO (1652 entries) or 1,3 EE (931 entries) are most favored. The other EO modes μ_3_ (159 entries) or μ_4_ (11 entries) are far less frequent. Similar observations are made for the more complex end-to-end bridging modes: μ_3_-1,1,3 (131), μ_4_-1,1,3,3 (13), μ_4_-1,1,1,3 (11), μ_5_-1,1,1,3,3 (1). For completeness, the occurrence of N_3_^−^ as a free anion is not so common, as only 92 entries were identified in the CSD database.

## Synthesis and crystallization   

The title compound was synthesized hydro­thermally under autogenous pressure from a mixture of iron(II) sulfate hepta­hydrate (28 mg, 0.1 mmol), quinolin-8-amine (15 mg, 0.1 mmol) and sodium azide NaN_3_ (13 mg, 0.2 mmol) in water–methanol (4:1 *v*/*v*, 20 ml). The mixture was sealed in a Teflon-lined autoclave and heated at 453 K for two days and cooled to room temperature at 10 K h^−1^. The crystals were obtained in *ca* 20% yield based on iron and proved to consist of a mononuclear heteroleptic Fe complex rather than the expected polymeric architecture with bridging azides.

CAUTION! Although not encountered in our experiments, azido compounds of metal ions are potentially explosive. Only a small amount of the materials should be prepared, and it should be handled with care.

## Refinement   

Crystal data, data collection and structure refinement details are summarized in Table 3 ▸. All H atoms were placed in geometrically idealized positions and constrained to ride on their parent atoms, with C—H distances of 0.93 Å, N—H distance of 0.89 Å and with 1.2*U*_eq_ of the parent atom.

## Supplementary Material

Crystal structure: contains datablock(s) global, I. DOI: 10.1107/S2056989016014808/pj2035sup1.cif

Structure factors: contains datablock(s) I. DOI: 10.1107/S2056989016014808/pj2035Isup2.hkl

CCDC reference: 1505176

Additional supporting information: crystallographic information; 3D view; checkCIF report

## Figures and Tables

**Table 1:** Selected geometric parameters (Å, °)

Fe1—N8	2.104 (3)	Fe1—N4	2.175 (2)
Fe1—N2	2.160 (3)	Fe1—N3	2.241 (3)
Fe1—N5	2.174 (3)	Fe1—N1	2.284 (3)
			
N8—Fe1—N2	91.14 (12)	N5—Fe1—N3	82.06 (11)
N8—Fe1—N5	94.16 (13)	N4—Fe1—N3	76.67 (9)
N2—Fe1—N5	95.49 (10)	N8—Fe1—N1	88.56 (13)
N8—Fe1—N4	94.82 (12)	N2—Fe1—N1	75.65 (10)
N2—Fe1—N4	167.88 (9)	N5—Fe1—N1	170.81 (10)
N5—Fe1—N4	94.59 (10)	N4—Fe1—N1	93.92 (9)
N8—Fe1—N3	170.31 (11)	N3—Fe1—N1	96.56 (10)
N2—Fe1—N3	98.09 (9)		

**Table 2:** Hydrogen-bond geometry (Å, °)

*D*—H⋯*A*	*D*—H	H⋯*A*	*D*⋯*A*	*D*—H⋯*A*
N1—H1*A*⋯N10^i^	0.89	2.62	3.361 (6)	141
N1—H1*B*⋯N10^ii^	0.89	2.42	3.254 (6)	157
N3—H3*A*⋯N7^i^	0.89	2.22	3.019 (4)	149
N3—H3*B*⋯N5^iii^	0.89	2.72	3.561 (4)	159

Symmetry codes: (i) 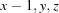; (ii) 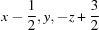; (iii) 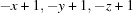.

**Table 3:** Experimental details

Crystal data	
Chemical formula	[Fe(N_3_)(C_9_H_8_N_2_)_2_]
*M*_r_	428.26
Crystal system, space group	Orthorhombic, *P* *b* *c* *a*
Temperature (K)	296
*a*, *b*, *c* (Å)	8.1798 (8), 15.8675 (13), 27.775 (4)
*V* (Å^3^)	3605.0 (6)
*Z*	8
Radiation type	Mo *K*α
μ (mm^−1^)	0.87
Crystal size (mm)	0.35 × 0.21 × 0.11

Data collection	
Diffractometer	Bruker–Nonius Kappa CCD with an APEXII detector
Absorption correction	Multi-scan (*SADABS*; Sheldrick, 2003 ▸)
*T*_min_, *T*_max_	0.606, 0.746
No. of measured, independent and observed [*I* > 2σ(*I*)] reflections	16951, 4100, 2081
*R*_int_	0.092
(sin θ/λ)_max_ (Å^−1^)	0.649

Refinement	
*R*[*F* ^2^ > 2σ(*F* ^2^)], *wR*(*F* ^2^), *S*	0.053, 0.121, 0.97
No. of reflections	4100
No. of parameters	262
H-atom treatment	H-atom parameters constrained
Δρ_max_, Δρ_min_ (e Å^−3^)	0.32, −0.36

Computer programs: *APEX2* and *SAINT* (Bruker, 2009 ▸), *SHELXT2014* (Sheldrick, 2015*a* ▸), *SHELXL2014* (Sheldrick, 2015*b* ▸), *Mercury* (Macrae *et al.*, 2008 ▸) and *publCIF* (Westrip, 2010 ▸).

## supplementary crystallographic information

### Crystal data

**Table:**

[Fe(N_3_)(C_9_H_8_N_2_)_2_]	*D*_x_ = 1.578 Mg m^−^^3^
*M**_r_* = 428.26	Mo *K*α radiation, λ = 0.71073 Å
Orthorhombic, *P**b**c**a*	Cell parameters from 1795 reflections
*a* = 8.1798 (8) Å	θ = 2.4–26.9°
*b* = 15.8675 (13) Å	µ = 0.87 mm^−^^1^
*c* = 27.775 (4) Å	*T* = 296 K
*V* = 3605.0 (6) Å^3^	Prism, red
*Z* = 8	0.35 × 0.21 × 0.11 mm
*F*(000) = 1760	

### Data collection

**Table:**

Bruker–Nonius Kappa CCD with an APEXII detector diffractometer	4100 independent reflections
Radiation source: fine focus sealed tube	2081 reflections with *I* > 2σ(*I*)
Graphite monochromator	*R*_int_ = 0.092
φ and ω scans	θ_max_ = 27.5°, θ_min_ = 2.9°
Absorption correction: multi-scan (SADABS; Sheldrick, 2003)	*h* = −10→9
*T*_min_ = 0.606, *T*_max_ = 0.746	*k* = −20→20
16951 measured reflections	*l* = −34→35

### Refinement

**Table:**

Refinement on *F*^2^	0 restraints
Least-squares matrix: full	Hydrogen site location: inferred from neighbouring sites
*R*[*F*^2^ > 2σ(*F*^2^)] = 0.053	H-atom parameters constrained
*wR*(*F*^2^) = 0.121	*w* = 1/[σ^2^(*F*_o_^2^) + (0.0475*P*)^2^] where *P* = (*F*_o_^2^ + 2*F*_c_^2^)/3
*S* = 0.97	(Δ/σ)_max_ < 0.001
4100 reflections	Δρ_max_ = 0.32 e Å^−^^3^
262 parameters	Δρ_min_ = −0.36 e Å^−^^3^

### Special details

**Table:**

Geometry. All esds (except the esd in the dihedral angle between two l.s. planes) are estimated using the full covariance matrix. The cell esds are taken into account individually in the estimation of esds in distances, angles and torsion angles; correlations between esds in cell parameters are only used when they are defined by crystal symmetry. An approximate (isotropic) treatment of cell esds is used for estimating esds involving l.s. planes.

### Fractional atomic coordinates and isotropic or equivalent isotropic displacement parameters (Å^2^)

**Table:**

	*x*	*y*	*z*	*U*_iso_*/*U*_eq_	
Fe1	0.50883 (5)	0.47368 (3)	0.61957 (2)	0.03352 (17)	
N1	0.3287 (3)	0.49268 (15)	0.68146 (12)	0.0413 (8)	
H1A	0.2316	0.4718	0.6733	0.050*	
H1B	0.3637	0.4649	0.7073	0.050*	
N2	0.4757 (3)	0.60857 (16)	0.62329 (10)	0.0327 (7)	
N3	0.3216 (3)	0.44621 (15)	0.56279 (11)	0.0341 (7)	
H3A	0.2237	0.4634	0.5728	0.041*	
H3B	0.3461	0.4745	0.5361	0.041*	
N4	0.4890 (3)	0.33703 (15)	0.62114 (10)	0.0309 (6)	
N5	0.6707 (3)	0.47625 (17)	0.55749 (12)	0.0482 (9)	
N6	0.8137 (3)	0.47003 (15)	0.56124 (12)	0.0387 (8)	
N7	0.9542 (3)	0.4642 (2)	0.56381 (16)	0.0735 (13)	
N8	0.7006 (4)	0.4793 (2)	0.66995 (14)	0.0680 (11)	
N9	0.8201 (4)	0.46219 (18)	0.68843 (14)	0.0522 (9)	
N10	0.9391 (5)	0.4460 (3)	0.70919 (18)	0.1184 (19)	
C1	0.3122 (4)	0.58115 (19)	0.69286 (14)	0.0337 (8)	
C2	0.2228 (4)	0.6099 (2)	0.73119 (14)	0.0439 (10)	
H2	0.1680	0.5719	0.7509	0.053*	
C3	0.2137 (4)	0.6972 (3)	0.74073 (15)	0.0512 (11)	
H3	0.1540	0.7162	0.7671	0.061*	
C4	0.2909 (4)	0.7538 (2)	0.71204 (15)	0.0458 (10)	
H4	0.2848	0.8110	0.7191	0.055*	
C5	0.3793 (4)	0.7268 (2)	0.67206 (14)	0.0359 (9)	
C6	0.4590 (4)	0.7814 (2)	0.64010 (15)	0.0408 (10)	
H6	0.4556	0.8392	0.6455	0.049*	
C7	0.5399 (4)	0.7512 (2)	0.60195 (15)	0.0444 (10)	
H7	0.5918	0.7878	0.5807	0.053*	
C8	0.5459 (4)	0.6630 (2)	0.59412 (14)	0.0417 (9)	
H8	0.6016	0.6429	0.5673	0.050*	
C9	0.3901 (3)	0.63909 (18)	0.66224 (12)	0.0277 (7)	
C10	0.3161 (3)	0.35638 (18)	0.55234 (12)	0.0276 (8)	
C11	0.2290 (4)	0.3235 (2)	0.51487 (13)	0.0378 (9)	
H11	0.1705	0.3591	0.4946	0.045*	
C12	0.2274 (4)	0.2361 (2)	0.50673 (15)	0.0460 (10)	
H12	0.1678	0.2145	0.4810	0.055*	
C13	0.3114 (4)	0.1829 (2)	0.53578 (15)	0.0437 (10)	
H13	0.3108	0.1253	0.5294	0.052*	
C14	0.3997 (4)	0.21408 (19)	0.57561 (14)	0.0330 (8)	
C15	0.4861 (4)	0.1629 (2)	0.60823 (14)	0.0427 (10)	
H15	0.4863	0.1047	0.6043	0.051*	
C16	0.5685 (4)	0.1980 (2)	0.64518 (16)	0.0493 (11)	
H16	0.6258	0.1643	0.6668	0.059*	
C17	0.5670 (4)	0.2858 (2)	0.65075 (15)	0.0437 (10)	
H17	0.6237	0.3091	0.6765	0.052*	
C18	0.4037 (3)	0.30219 (19)	0.58328 (12)	0.0280 (8)	

### Atomic displacement parameters (Å^2^)

**Table:**

	*U*^11^	*U*^22^	*U*^33^	*U*^12^	*U*^13^	*U*^23^
Fe1	0.0268 (2)	0.0307 (3)	0.0430 (3)	0.0016 (2)	−0.0010 (3)	−0.0070 (2)
N1	0.0433 (17)	0.0335 (16)	0.047 (2)	0.0018 (13)	0.0047 (16)	0.0042 (14)
N2	0.0287 (14)	0.0356 (15)	0.0339 (18)	−0.0009 (11)	0.0033 (15)	−0.0009 (14)
N3	0.0251 (13)	0.0356 (15)	0.042 (2)	0.0024 (11)	0.0027 (14)	0.0039 (14)
N4	0.0314 (13)	0.0311 (14)	0.0302 (17)	0.0058 (12)	−0.0011 (15)	−0.0012 (13)
N5	0.0304 (15)	0.061 (2)	0.054 (2)	−0.0014 (14)	0.0045 (15)	−0.0090 (17)
N6	0.0358 (16)	0.0280 (15)	0.052 (2)	−0.0033 (13)	0.0083 (15)	−0.0075 (14)
N7	0.0288 (16)	0.073 (2)	0.119 (4)	0.0023 (15)	0.007 (2)	−0.026 (2)
N8	0.0478 (19)	0.094 (3)	0.062 (3)	0.0134 (19)	−0.025 (2)	−0.020 (2)
N9	0.0447 (19)	0.051 (2)	0.061 (3)	0.0080 (16)	−0.0083 (19)	−0.0333 (18)
N10	0.084 (3)	0.155 (4)	0.116 (4)	0.068 (3)	−0.053 (3)	−0.080 (3)
C1	0.0308 (17)	0.0343 (19)	0.036 (2)	0.0068 (14)	0.0010 (17)	0.0049 (17)
C2	0.0412 (19)	0.058 (2)	0.033 (2)	0.0112 (18)	0.0085 (19)	0.010 (2)
C3	0.054 (2)	0.065 (3)	0.034 (3)	0.025 (2)	0.006 (2)	−0.004 (2)
C4	0.051 (2)	0.041 (2)	0.045 (3)	0.0187 (18)	−0.006 (2)	−0.008 (2)
C5	0.0359 (18)	0.040 (2)	0.032 (2)	0.0067 (15)	−0.0071 (18)	−0.0013 (18)
C6	0.044 (2)	0.0315 (18)	0.047 (3)	−0.0025 (15)	−0.012 (2)	0.0004 (19)
C7	0.043 (2)	0.038 (2)	0.052 (3)	−0.0060 (16)	−0.001 (2)	0.013 (2)
C8	0.044 (2)	0.047 (2)	0.034 (2)	−0.0027 (16)	0.0071 (18)	0.0066 (19)
C9	0.0254 (15)	0.0319 (18)	0.026 (2)	0.0081 (13)	−0.0032 (15)	−0.0002 (16)
C10	0.0241 (15)	0.0314 (17)	0.027 (2)	−0.0040 (13)	0.0042 (15)	−0.0001 (16)
C11	0.0338 (18)	0.051 (2)	0.029 (2)	−0.0030 (16)	−0.0022 (17)	−0.0013 (19)
C12	0.043 (2)	0.058 (3)	0.037 (3)	−0.0142 (19)	−0.001 (2)	−0.014 (2)
C13	0.041 (2)	0.038 (2)	0.052 (3)	−0.0121 (17)	0.013 (2)	−0.012 (2)
C14	0.0307 (17)	0.0318 (19)	0.036 (2)	−0.0013 (14)	0.0095 (17)	−0.0008 (17)
C15	0.0441 (19)	0.0313 (18)	0.053 (3)	−0.0008 (17)	0.012 (2)	0.0004 (17)
C16	0.050 (2)	0.040 (2)	0.057 (3)	0.0112 (17)	0.000 (2)	0.014 (2)
C17	0.0431 (19)	0.046 (2)	0.042 (3)	0.0032 (17)	−0.0066 (19)	0.001 (2)
C18	0.0236 (15)	0.0353 (18)	0.025 (2)	−0.0013 (14)	0.0085 (15)	−0.0023 (16)

### Geometric parameters (Å, º)

**Table:**

Fe1—N8	2.104 (3)	C3—H3	0.9300
Fe1—N2	2.160 (3)	C4—C5	1.392 (5)
Fe1—N5	2.174 (3)	C4—H4	0.9300
Fe1—N4	2.175 (2)	C5—C6	1.401 (5)
Fe1—N3	2.241 (3)	C5—C9	1.421 (4)
Fe1—N1	2.284 (3)	C6—C7	1.338 (5)
N1—C1	1.445 (4)	C6—H6	0.9300
N1—H1A	0.8900	C7—C8	1.416 (5)
N1—H1B	0.8900	C7—H7	0.9300
N2—C8	1.316 (4)	C8—H8	0.9300
N2—C9	1.377 (4)	C10—C11	1.365 (4)
N3—C10	1.455 (4)	C10—C18	1.411 (4)
N3—H3A	0.8900	C11—C12	1.405 (4)
N3—H3B	0.8900	C11—H11	0.9300
N4—C17	1.321 (4)	C12—C13	1.355 (5)
N4—C18	1.378 (4)	C12—H12	0.9300
N5—N6	1.179 (3)	C13—C14	1.411 (5)
N6—N7	1.155 (3)	C13—H13	0.9300
N8—N9	1.136 (4)	C14—C15	1.407 (5)
N9—N10	1.160 (4)	C14—C18	1.415 (4)
C1—C2	1.370 (4)	C15—C16	1.349 (5)
C1—C9	1.405 (4)	C15—H15	0.9300
C2—C3	1.412 (5)	C16—C17	1.401 (4)
C2—H2	0.9300	C16—H16	0.9300
C3—C4	1.357 (5)	C17—H17	0.9300
			
N8—Fe1—N2	91.14 (12)	C3—C4—C5	120.4 (3)
N8—Fe1—N5	94.16 (13)	C3—C4—H4	119.8
N2—Fe1—N5	95.49 (10)	C5—C4—H4	119.8
N8—Fe1—N4	94.82 (12)	C4—C5—C6	123.8 (3)
N2—Fe1—N4	167.88 (9)	C4—C5—C9	119.1 (3)
N5—Fe1—N4	94.59 (10)	C6—C5—C9	117.0 (3)
N8—Fe1—N3	170.31 (11)	C7—C6—C5	120.7 (3)
N2—Fe1—N3	98.09 (9)	C7—C6—H6	119.6
N5—Fe1—N3	82.06 (11)	C5—C6—H6	119.6
N4—Fe1—N3	76.67 (9)	C6—C7—C8	119.5 (3)
N8—Fe1—N1	88.56 (13)	C6—C7—H7	120.3
N2—Fe1—N1	75.65 (10)	C8—C7—H7	120.3
N5—Fe1—N1	170.81 (10)	N2—C8—C7	122.6 (3)
N4—Fe1—N1	93.92 (9)	N2—C8—H8	118.7
N3—Fe1—N1	96.56 (10)	C7—C8—H8	118.7
C1—N1—Fe1	110.7 (2)	N2—C9—C1	118.4 (3)
C1—N1—H1A	109.5	N2—C9—C5	121.8 (3)
Fe1—N1—H1A	109.5	C1—C9—C5	119.8 (3)
C1—N1—H1B	109.5	C11—C10—C18	119.8 (3)
Fe1—N1—H1B	109.5	C11—C10—N3	122.8 (3)
H1A—N1—H1B	108.1	C18—C10—N3	117.4 (3)
C8—N2—C9	118.3 (3)	C10—C11—C12	120.3 (3)
C8—N2—Fe1	124.5 (2)	C10—C11—H11	119.8
C9—N2—Fe1	116.8 (2)	C12—C11—H11	119.8
C10—N3—Fe1	110.59 (18)	C13—C12—C11	121.0 (3)
C10—N3—H3A	109.5	C13—C12—H12	119.5
Fe1—N3—H3A	109.5	C11—C12—H12	119.5
C10—N3—H3B	109.5	C12—C13—C14	120.5 (3)
Fe1—N3—H3B	109.5	C12—C13—H13	119.7
H3A—N3—H3B	108.1	C14—C13—H13	119.7
C17—N4—C18	118.2 (3)	C15—C14—C13	124.0 (3)
C17—N4—Fe1	126.2 (2)	C15—C14—C18	117.5 (3)
C18—N4—Fe1	115.0 (2)	C13—C14—C18	118.5 (3)
N6—N5—Fe1	122.2 (3)	C16—C15—C14	120.2 (3)
N7—N6—N5	178.5 (5)	C16—C15—H15	119.9
N9—N8—Fe1	158.7 (3)	C14—C15—H15	119.9
N8—N9—N10	177.0 (5)	C15—C16—C17	119.3 (3)
C2—C1—C9	119.7 (3)	C15—C16—H16	120.3
C2—C1—N1	122.9 (3)	C17—C16—H16	120.3
C9—C1—N1	117.4 (3)	N4—C17—C16	123.2 (4)
C1—C2—C3	120.0 (3)	N4—C17—H17	118.4
C1—C2—H2	120.0	C16—C17—H17	118.4
C3—C2—H2	120.0	N4—C18—C10	118.5 (3)
C4—C3—C2	121.0 (4)	N4—C18—C14	121.5 (3)
C4—C3—H3	119.5	C10—C18—C14	119.9 (3)
C2—C3—H3	119.5		
			
Fe1—N1—C1—C2	−174.2 (3)	Fe1—N3—C10—C11	−171.0 (2)
Fe1—N1—C1—C9	6.9 (3)	Fe1—N3—C10—C18	9.9 (3)
C9—C1—C2—C3	−2.1 (5)	C18—C10—C11—C12	−0.5 (5)
N1—C1—C2—C3	179.0 (3)	N3—C10—C11—C12	−179.6 (3)
C1—C2—C3—C4	0.9 (5)	C10—C11—C12—C13	0.1 (5)
C2—C3—C4—C5	0.8 (6)	C11—C12—C13—C14	1.4 (5)
C3—C4—C5—C6	178.4 (3)	C12—C13—C14—C15	178.1 (3)
C3—C4—C5—C9	−1.2 (5)	C12—C13—C14—C18	−2.5 (5)
C4—C5—C6—C7	−179.0 (3)	C13—C14—C15—C16	179.5 (3)
C9—C5—C6—C7	0.6 (5)	C18—C14—C15—C16	0.1 (5)
C5—C6—C7—C8	−0.5 (5)	C14—C15—C16—C17	0.1 (5)
C9—N2—C8—C7	1.6 (5)	C18—N4—C17—C16	0.7 (5)
Fe1—N2—C8—C7	−170.7 (2)	Fe1—N4—C17—C16	−169.8 (3)
C6—C7—C8—N2	−0.6 (5)	C15—C16—C17—N4	−0.5 (6)
C8—N2—C9—C1	178.7 (3)	C17—N4—C18—C10	178.1 (3)
Fe1—N2—C9—C1	−8.4 (3)	Fe1—N4—C18—C10	−10.3 (3)
C8—N2—C9—C5	−1.5 (4)	C17—N4—C18—C14	−0.5 (4)
Fe1—N2—C9—C5	171.4 (2)	Fe1—N4—C18—C14	171.1 (2)
C2—C1—C9—N2	−178.4 (3)	C11—C10—C18—N4	−179.3 (3)
N1—C1—C9—N2	0.5 (4)	N3—C10—C18—N4	−0.2 (4)
C2—C1—C9—C5	1.8 (5)	C11—C10—C18—C14	−0.6 (4)
N1—C1—C9—C5	−179.3 (3)	N3—C10—C18—C14	178.5 (3)
C4—C5—C9—N2	−180.0 (3)	C15—C14—C18—N4	0.1 (4)
C6—C5—C9—N2	0.4 (4)	C13—C14—C18—N4	−179.3 (3)
C4—C5—C9—C1	−0.1 (5)	C15—C14—C18—C10	−178.5 (3)
C6—C5—C9—C1	−179.7 (3)	C13—C14—C18—C10	2.1 (4)

### Hydrogen-bond geometry (Å, º)

**Table:**

*D*—H···*A*	*D*—H	H···*A*	*D*···*A*	*D*—H···*A*
N1—H1*A*···N10^i^	0.89	2.62	3.361 (6)	141
N1—H1*B*···N10^ii^	0.89	2.42	3.254 (6)	157
N3—H3*A*···N7^i^	0.89	2.22	3.019 (4)	149
N3—H3*B*···N5^iii^	0.89	2.72	3.561 (4)	159

Symmetry codes: (i) *x*−1, *y*, *z*; (ii) *x*−1/2, *y*, −*z*+3/2; (iii) −*x*+1, −*y*+1, −*z*+1.
